# Associations of MTHFR C677T polymorphism with insulin resistance, results of NURSE Study (Nursing Unacquainted Related Stress Etiologies)

**DOI:** 10.1186/s40200-017-0303-9

**Published:** 2017-05-22

**Authors:** Motahareh Kheradmand, Zhila Maghbooli, Sedigheh Salemi, Mahnaz Sanjari

**Affiliations:** 1 Health Science Research Center, Mazandaran University of Medical Science, Sari, Iran; 2 Osteoporosis Research Center, Endocrinology and Metabolism Clinical Sciences Institute, Tehran University of Medical Sciences, Tehran, Iran; 3 School of Nursing and Midwifery, Iran University of Medical Sciences, Tehran, Iran; 4 Diabetes Research Center, Endocrinology and Metabolism Clinical Sciences Institute, Tehran University of Medical Sciences, Tehran, Iran; 5 Endocrinology and Metabolism Research Center, Endocrinology and Metabolism Clinical Sciences Institute, Tehran University of Medical Sciences, Tehran, Iran

**Keywords:** Insulin Resistance, MTHFR, C677T Polymorphism

## Abstract

**Background:**

The insulin resistance syndrome is one of the major contributors of metabolic syndrome, diabetes Type 2 and atherosclerotic cardiovascular disease. A common mutation (677C to T; Ala to Val) in the methylenetetrahydrofolate reductase (MTHFR) gene is associated with decreased specific MTHFR activity and elevation of the homocysteine. The aim of this study is investigation of association between MTHFR 677C > T polymorphism with insulin resistance by using HOMA (Homeostasis Model Assessment) index in nurses who are potentially prone to develop insulin resistance because of unfavorable effects of shift work.

**Method:**

Nursing Unacquainted Related Stress Etiologies Study (Nurse Study) was conducted in five different educational hospitals of Tehran University of Medical Science (TUMS). The nurses aged 22–57 who have been referred by the matron were recruited. A self-administered questionnaire was completed. Anthropometric measurements including weight, height, waist and hip circumference in addition to blood pressure were measured. Insulin resistance and Insulin sensitivity were measured using the homeostatic model assessment (HOMA) and quantitative insulin sensitivity check index (QUICKI) respectively. The detection of MTHFR C677T polymorphism in exon four of MTHFR gene was performed by polymerase chain reaction–restriction fragment length polymorphism (PCR–RFLP) analysis using HINFI restriction enzyme digestion.

**Result:**

A total of 273 subjects were recruited in the study. CT genotype were detected in 51.6% (129) subjects and CC and TT genotype were seen in 9.2% (25) and 35.2% (96) subjects respectively. Participants with TT genotype (9.65 ± 4.00) have significantly lower insulin level than participants with CT genotype (14.12 ± 15.34) (*p*-value: 0.01). The same significant difference was observed for HOMA index (*p*-value: 0.03). Result showed that HOMA is lower in subjects who are taking supplements.

**Conclusion:**

Result of this study showed subjects with TT genotype had significantly lower HOMA compare to CT genotype and the same pattern was seen for insulin level. We also found subjects taking supplement have lower HOMA compared to others regardless of their genotype.

## Background

The insulin resistance syndrome is characterized by glucose intolerance, hyperinsulinaemia, dyslipidemia, abdominal obesity and hypertension [1] Insulin resistance is a fundamental abnormality in the pathogenesis of type 2 diabetes and atherosclerotic cardiovascular disease (CVD) [1–3] Insulin resistance disturbs the functions of insulin target organs, such as adipose tissue, which is related to glucose metabolism, lipogenesis, and adipokine secretion [4]. Moreover insulin resistance is one of the major contributors of metabolic syndrome although the underlying mechanisms are not clear [5].

Insulin resistance affected by different sets of genetic and environmental factors. The set of genetic which influences all the components may initiate the abnormalities of insulin resistance [6]. There is considerable evidence which supports a genetic basis for insulin resistance. MTHFR is one of the key enzymes in the process of folic acid metabolism, which is an important substrate for DNA synthesis. It has a major role in DNA methylation and has a close association with the synthesis of methionine [7]. Genetic mutations in MTHFR are the most commonly known inherited risks factor for elevated homocysteine levels [8]. A common mutation (677C to T; Ala to Val) in the MTHFR gene is associated with decreased specific MTHFR activity and elevation of the homocysteine [9]. This polymorphism impairs the regulation of homocysteine and adequate folate levels [8].

Hyperhomocysteinemia is considered as a risk factor for atherothrombotic disease independent of other conventional risk factors [10]. A meta-analysis revealed the association of homocysteine (Hcy) with coronary heart disease in the Middle East and in Asian countries [11]. The association between insulin resistance and plasma homocysteine levels are debated [12]. Additionally, mechanisms for a putative association [13] and the direction of causality in this association are not clear [14]. Animal studies suggest a possible role of insulin in the regulation of plasma Hcy concentration by affecting the hepatic transsulfuration pathway [14]. Also, plasma levels of insulin seem to influence homocysteine metabolism, possibly through effects on glomerular filtration or by influencing activity of key enzymes in homocysteine metabolism, including 5, 10-methylenetetrahydrofolate reductase (MTHFR) or cystathione b-synthase (CBS) [2]. Little is known about the relationship between homocysteine and insulin sensitivity in humans [12]. Folate decreases homocysteine levels by increasing the rate of recycling of homocysteine to methionine. It is assumed that folic acid affect endothelium and therefore could directly improve endothelial function [15]. Beneficial effects of folic acid supplementation have been reported in patient with unstable angina and Hyperhomocysteinemia [16], in patients with metabolic syndrome [17] and in healthy overweight subjects [18].

The literature has been suggested that existence of significant gene-environment interaction effects make some individuals more susceptible to cardiovascular diseases [19]. Night shift workers are indicated to be associated with adverse metabolic disorders. It has been reported that shift work is significantly associated with insulin resistance and Metabolic Syndrome (MetS) [20–22]. short sleep duration in night and desynchronization of circadian rhythm would be two major aspects of night shift work [2] that were found to disturb metabolism [3–5] and impair components of MetS [6, 7], which may consequently result in the occurrence of MetS among night shift workers. The impact of shift work on MetS is not yet completely understood, but workers involved in night shifts may be at increased risk, because of unfavorable effects of sleep deprivation on the main components MetS [22]. The nurses who work at high risk environment are one of the target group to be assesed for personalized risk prediction and strategic health-care planning which can facilitate a new form of preventive care.

According to our knowledge only few studies have investigated the effect of MTHFR 677C > T polymorphism with insulin resistance [23, 24]. It seems recognition of insulin resistance for identifying subjects at high risk of diabetes T2 and/or cardiovascular disease is clinically relevant homeostasis [25]. Regarding the importance and efficacy of prevention strategies by identifying susceptible population, this present study aimed to investigate the association between MTHFR 677C > T polymorphism with insulin resistance by using HOMA index in nurses who are potentially prone to develop insulin resistance because of unfavorable effects of shift work.

## Method

### Subjects

Nursing Unacquainted Related Stress Etiologies (NURSE) Study was conducted in five different educational hospitals of Tehran University of Medical Science (TUMS). The nurses aged 22–57 who have been referred by the matron in cooperation with Iranian Nursing Organization (INO) were recruited. All nurses in these hospitals who desire to attend in the study were eligible. Exclusion criteria were: history of any condition chronic diseases such as known cardiovascular diseases, thyroid diseases, malignancies, current smoking, diabetes mellitus, heart failure, acute or chronic infections, and hepatic or renal diseases. Moreover, any subjects who had a positive history of smoking were excluded from the study.

### Data collection

A self-administered questionnaire consisted demographic information, educational level, medical history, medicine, supplements intake and types of work shift were completed by participants of the study. The educational level was classified as bachelor's science degree (Bs), master degree (Ms) and doctorate degree (PhD). Anthropometric measurement included weight, height, waist and hip circumference and also blood pressure were measured. Trained laboratory technicians obtained fasting (10–12 h) venous blood samples and levels of total cholesterol, triglyceride (TG), high-density lipoprotein cholesterol (HDL-C), LDL-C, fasting blood sugar (FBS) and insulin were determined. Insulin resistance and Insulin sensitivity were measured by t homeostatic model assessment (HOMA) and quantitative insulin sensitivity check index (QUICKI) respectively. Serum insulin was also measured using Immunoenzymometric assay, Monobind INC, USA technique.

### DNA extraction

The peripheral blood mononuclear cells (PBMCs) were separated from whole blood through density gradient centrifugation using Ficoll (Lympholyte, Cell Separation Media). Genomic DNA was isolated from PBMCs by using QIAamp DNA mini kit.

### Genotyping of MTHFR C677T polymorphism

**Fig. 1:**
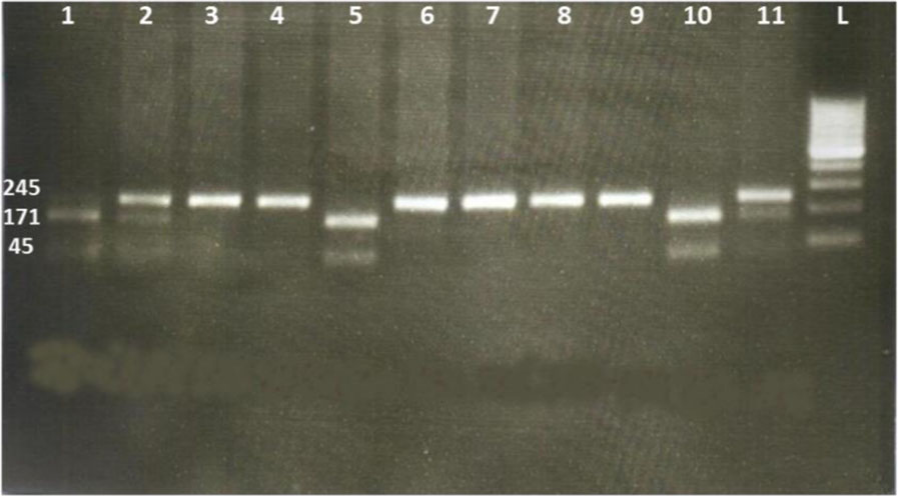
Gel electrophoresis showing PCR- RFLP analysis of MTHFR, C677T locus; rs1801133. Detection of C677T polymorphism after HinfI digestion: fragments in lanes 1, 5, and 10 (171bp, 74 bp) indicate homozygote (CC) genotype; 3,4, 6, 7, 8, and 9 (245 bp) lanes 1, 5 and 10 showing a single band (245 bp) of undigested PCR product indicates homozygote (TT) genotype; lanes 2,and 11 heterozygote (C/T) genotype (245, 171 and 74 bp). L; ladder 100bp, gel 2%

**Fig. 2:**
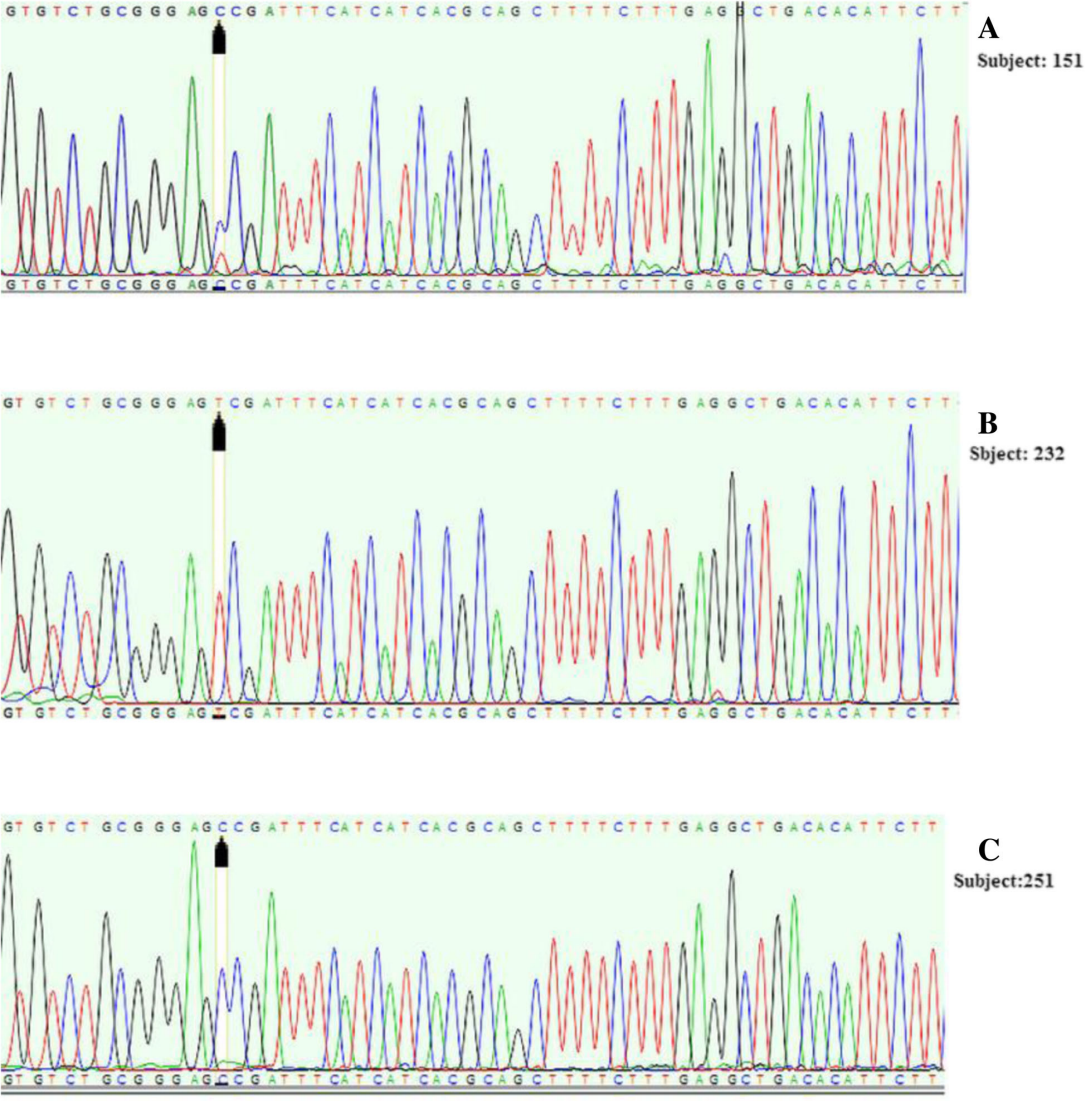
Sequencing results of MTHFR, C677T locus; rs1801133. Detection of C677T polymorphism by sequencing: **a**, heterozygote (C/T) genotype; **b**, homozygote (TT) genotype, **c**, homozygote (CC) genotype

The detection of MTHFR C677T polymorphism in exon four of MTHFR gene was performed by polymerase chain reaction–restriction fragment length polymorphism (PCR–RFLP) analysis using HINFI restriction enzyme digestion as previously described by Frosst et al [26]. The PCR forward and reverse primers were5′-AGG ACG GTG CGG TGA GAG TG -3′ and 5′-CAAAGG CCA CCC CGA AGC -3′, respectively. Existence of the C677T mutation in the PCR product introduced a new HINFI restriction site, which resulted in the digestion of the 246-bp amplicon into 175 and 71-bp fragments. Digested DNA fragments were then visualized on a 2% agarose gel (Fig. 1). The validity of the PCR–RFLP MTHFR genotype was confirmed by sequencing the 15% of total PCR products (Fig. 2).

### Statistical analysis

Data were analyzed by the SPSS 19 package program, to obtain Mean ± SD values, Student’s *T*-test, analysis of variance and chi-square tests, as appropriate.

Student’s *T* test and analysis of variance (ANOVA) followed by Bonferroni test were used to determine differences in means, and intergroup significance was assessed by chi-square test.

The Homeostasis Model Assessment of IR (HOMA-IR) was calculated according to the formula: fasting insulin (microU/mL) x fasting glucose (mg/dL)/405. The quantitative insulin sensitivity check index (QUICKI) is derived using the inverse of the sum of the logarithms of the fasting insulin and fasting glucose: 1/(log(fasting insulin microU/mL) + log(fasting glucose mg/dL)). HOMA-IR and anthropometric measurements were categorized into quartiles. The threshold HOMA-IR level to define insulin resistance was made on the 75th percentile (≥2.93) of values in the study population. Effect sizes were expressed as odds ratios with accompanying 95% confidence intervals. The significance level used throughout the study was set at 0.05.

## Results

A total of 273 subjects were recruited in the study. The study subjects’ characteristics are shown in Table 1 in which the mean of age was 35.01 ± 6.52.

**Table 1:** The characteristic of study subjects (*n* = 273)

	*N*(%)
Age (mean ± sd)	35.01 ± 6.52
Sex (female)	246(91.1)
BMI ≥ 30	31 (11.5)
FBS ≥ 100	30 (11.2)
TG ≥ 150	39 (14.5)
HDL < 40 men	9 (37.5)
HDL < 50 women	85 (84.8)
waist circumference ≥ 94 men	14 (58.3)
waist circumference ≥80 women	12 (4.9)
Hip circumference	94.69 ± 9.9
Folic Acid intake	315.80 ± 128.53
Vit B12 intake	2.65 ± 1.44

**Fig. 3:**
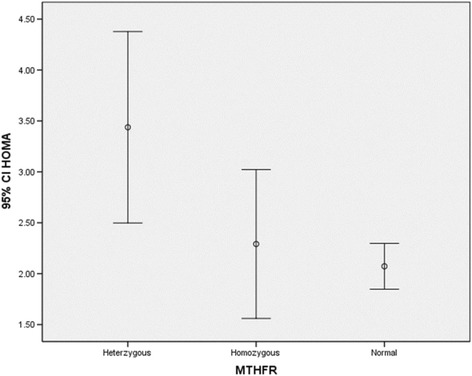
HOMA index in different genotype groups. The subjects with a genotype that carrying T allele (TT or CT) had significantly higher HOMA index than CC *genotype*. The CC genotype is referred to as “*homozygous normal*,” and the TT *genotype* as the“

**Fig. 4:**
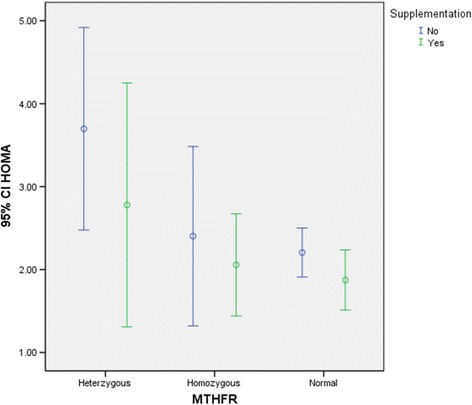
HOMA index in different genotype groups based on taking supplement. The result showed That HOMA is lower in subjects who are taking supplements such as folic acid and vitamin B12 and this pattern is consistent in all genotypes groups. The CC *genotype* is referred to as “*homozygous normal*,” and the TT *genotype* as the “*homozygous variant*.” The CT *genotype* is described as “*heterozygous*”

The genotype distributions of the MTHFR 677C > T polymorphism did not deviate from the Hardy–Weinberg equilibrium (*p* = 0.3). CT genotype were detected in 51.6% (129) subjects and CC and TT genotype were seen in 9.2% (25) and 35.2%(96) subjects respectively (Figs. 3 and 4).

The mean of FBS, HOMA and QUICKI in different genotype groups were analyzed using ANOVA as shown in Table 2. Bonferroni test showed the mean of insulin differ significantly between CT genotype and TT genotype. Subjects with TT genotype and CT genotype had significantly higher insulin level than subjects with CC genotype. Also, subjects with a genotype that carrying T allele (TT or CT) had significantly higher HOMA index than CC genotype (Fig. 3). We also checked the effect of supplement intake (folic acid and vitamin B12) on HOMA and QUCKI index. Result showed HOMA is lower in subjects who are taking supplements (Fig. 4).this pattern is consistent in all genotypes groups.

**Table 2:** Insulin level, FBS, HOMA and QUICKI in different genotype groups

	C T(129)	C C (25)	T T (96)	*P*-value
	Mean ± sd	Mean ± sd	Mean ± sd	
Insulin	14.12 ± 15.43	10.61 ± 5.60	9.65 ± 4.00	0.01
FBS	88.10 ± 24.03	83.92 ± 14.09	86.46 ± 10.36	0.55
HOMA	3.42 ± 5.40	2.29 ± 1.77	2.80 ± 4.02	0.03
QUICKI	0.34 ± 0.04	0.35 ± 0.03	0.35 ± 0.03	0.15

*FBS* fasting blood sugar, *HOMA* homeostatic model assessment, *QUICKI* quantitative insulin sensitivity check index

The effect of anthropometric variables on glucose profile is summarized in Tables 2 and 3. The relationship between glucose profile variation and age quartiles is shown in Table 2. In addition glucose profile variation in relation to BMI quartiles is shown in Table 3. All of glucose profile components including HOMA-IR, QUICKI, insulin and FBS were significantly different in BMI quartiles is shown in Table 4. In the Univariate analysis after fixing age, sex and BMI, there is a significant association between T allele and HOMA-IR (*p* = 0.02). The threshold HOMA-IR level to define insulin resistance was made on the 75th percentile (≥2.93) of values in the study population. After adjusting for BMI, there was a significant association between carrying T allele (TT or CT genotypes) and insulin resistance (*p* = 0.4); Odds ratio was 2.21 with 95% confidence interval between 1.03 and 4.75. Also, relative risk for this relationship was 1.35 with 95% confidence interval between 1.01 and 1.8.

**Table 3:** Glucose profile variation in relation to age quartiles

Glucose profile	Age Quartiles				
	1 (Mean ± SE)	2 (Mean ± SE)	3 (Mean ± SE)	4 (Mean ± SE)	*P*-value
HOMA-IR	1.93 ± 0.13	3.25 ± 0.52	2.81 ± 0.53	2.96 ± 0.60	0.2
QUICKI	0.34 ± 0.003	0.34 ± 0.005	0.35 ± 0.003	0.34 ± 0.004	0.1
Insulin (microU/mL)	9.33 ± 0.6	13.59 ± 1.6	12.06 ± 1.7	12.19 ± 1.4	0.1
FBS (mg/dL)	83.58 ± 1.26	90.59 ± 2.83	87.76 ± 2.06	86.89 ± 2.39	0.1

**Table 4:** Glucose profile variation in relation to BMI quartiles

Glucose profile	BMI Quartiles				
	1 (Mean ± SE)	2 (Mean ± SE)	3 (Mean ± SE)	4 (Mean ± SE)	*P*-value
HOMA-IR	1.80 ± 0.09	2.10 ± 0.18	2.17 ± 0.18	4.96 ± 0.86	0.01
QUICKI	0.35 ± 0.003	0.35 ± 0.004	0.35 ± 0.004	0.32 ± 0.004	0.01
Insulin (microU/mL)	9.11 ± 0.55	9.93 ± 0.74	10.25 ± 0.82	18.42 ± 2.35	0.01
FBS (mg/dL)	81.70 ± 1.26	83.96 ± 1.39	85.44 ± 1.49	98.01 ± 3.55	0.01

## Discussion

Results of this study showed an association between MTHFR 677C > T polymorphism with HOMA and serum insulin level. it demonstrated that an association between MTHFR 677C > T polymorphism with HOMA and serum insulin level. Subjects carrying T allele had significantly higher HOMA compare to CC genotype and the same pattern was seen for insulin level. This is the first study to find an association between MTHFR 677C > T polymorphism with insulin resistance in Iranian populations. This relationship has been reported in Chinese population [23] and obese adolescents [24]. Yang et al also showed that the MTHFR 677 T allele carriers had an increased risk of MetS [27]. Results of study performed on human hepatocyte cell line showed in presence of insulin and glucose, MTHFR and cystathionine-β-synthase activity decrease. These enzymes are responsible for remethylation and transsulfuration reactions respectively and maintain the plasma homocysteine Concentrations [28]. Although the association between Hhcy and C677T polymorphism in the MTHFR gene is inconsistent [13, 14, 29, 30]. Results of in vivo studies suggested Hhcy could be a factor causing insulin resistance [4, 28]. The study of Lee et al showed that Hcy promotes insulin resistance by inducing adipose endoplasmic reticulum stress and downstream inflammation in mice. It also has been suggested that Hhcy may impair insulin secretion, inhibit insulin signaling, as well as lead to endothelial dysfunction, contributing to insulin resistance [29]. On the other hand association of MTHFR C677T polymorphism with hypertension [31] the risk of MetS in Greek population [32] among ischemic stroke patients [33] and schizophrenia patients [34] has been reported. In addition this alteration seems to be associated with the carcinogenesis of the colorectum [7].

The results of the studies on enzyme activities have been shown that enzyme activity of C677T heterozygotes and homozygous variant (TT) is 65% and 30% respectively compared with common homozygotes variant (CC). From the microbiologic assay red cell folate level as well as plasma folate level is lower in heterozygotes and TT homozygotes compared with CC homozygotes. T allele of the 677C/T (A222V) MTHFR polymorphism causes a thermolability of the enzyme, reduces its activity, and inhibits the formation of 5-methyltetrahydrofolate, which serves as a methyl donor during the remethylation of homocysteine to methionine. This explains why TT homozygotes exhibit higher plasma homocysteine concentrations than CT heterozygotes and CC homozygotes in a majority of studies [35]

The interesting result of our study was the effect of supplement intake on insulin resistance. Our results showed subjects who are taking supplement have lower HOMA compare to others regardless of their genotype. There are few studies regarding the effect of folate in healthy subjects. Previous studies have shown the effect of prolonged folate treatment on homocysteine levels, insulin levels and improving insulin resistance in patients with metabolic syndrome [5] and patients with diabetes T2 [36]. Solini et al also reported healthy Subjects receiving folic acid supplementation showed a decrement of homocysteine and an amelioration of insulin sensitivity along with significant decrease in monocyte chemoattractant protein-1, interleukin-8 and C-reactive protein. These finding are in favor of controversial relationship between insulin and homocysteine Although the mechanisms by which folic acid decreases blood glucose concentration are not clearly understood, several hypotheses have been suggested. The possible mechanism of this relation is the active form of Hcy, may inhibit the insulin-stimulated tyrosine phosphorylation of insulin receptor β-subunit and its substrates and decrease the p85 regulatory subunit of phosphatidylinositol 3-kinase activity, including a reduction in insulin-stimulated glycogen synthesis. This naturally leads to insulin resistance and blood glucose increase. Another possible mechanism is that folic acid will ameliorate endothelial dysfunction induced by elevated Hcy, convert L-arginine to nitric oxide and L-citrulline, scavenge reactive oxygen species such as O2− and peroxynitrite, maintain a coupled endothelial nitric oxide synthase reaction, and prevent nitric oxide synthase dysfunction. All of these may be beneficial to glycomtabolism [36]. The nurses are considered as a target group to be assessed for personalized risk prediction and strategic health-care planning which can facilitate a new method of preventive care. Regarding the result of this study receiving folic acid can be a regarded as a preventive strategy.

The following limitations should be considered. Since our population was the young and healthy subjects, we did not consider metabolic syndrome as the outcome rather we define insulin resistance. We also did not measure homocysteine level in this study. Regardless of controversial relationship between Hcy and insulin resistance result of this study showed a significant association between MTHFR polymorphism with insulin resistance. Further studies need to be conducted to clarify the direction and mechanism of the association between Hcy and insulin resistance.

## Conclusion

Results of this study showed an association between MTHFR 677C > T polymorphism with HOMA and serum insulin level. The interesting result of this study was the effect of supplement intake on insulin resistance. Consuming the supplement specifically folic acid can be a regarded as a preventive strategy. In public health point of view identifying of high risk subjects and implementing preventive strategies are important. Therefore applying strategies in nurses who are potentially susceptible to develop metabolic syndrome and other related diseases are highly recommended.

## Abbreviations

CBS: cystathionine b-synthase
Hcy: Homocysteine
HOMA: Homeostatic model assessment
MetS: Metabolic syndrome
MTHFR: Methylenetetrahydrofolate
Nurse Study: Nursing unacquainted related stress etiologies study
PCR–RFLP: Polymerase chain reaction–restriction fragment length polymorphism
QUICKI: Quantitative insulin sensitivity check index
TUMS: Tehran University of Medical Science.
